# Private Hospital Workflow Optimization via Secure *k*-Means Clustering

**DOI:** 10.1007/s10916-019-1473-4

**Published:** 2019-11-29

**Authors:** Gabriele Spini, Maran van Heesch, Thijs Veugen, Supriyo Chatterjea

**Affiliations:** 1 Unit ICT, TNO, The Hague, The Netherlands; 2 Department of Cryptology, CWI, Amsterdam, The Netherlands; 3 Data Science Group, Philips Research, Eindhoven, The Netherlands

**Keywords:** Secure multi-party computation, Hospital, Workflow optimization, Privacy, Real-time locating system, Clustering, *k*-means

## Abstract

Optimizing the workflow of a complex organization such as a hospital is a difficult task. An accurate option is to use a real-time locating system to track locations of both patients and staff. However, privacy regulations forbid hospital management to assess location data of their staff members. In this exploratory work, we propose a secure solution to analyze the joined location data of patients and staff, by means of an innovative cryptographic technique called Secure Multi-Party Computation, in which an additional entity that the staff members can trust, such as a labour union, takes care of the staff data. The hospital, owning location data of patients, and the labour union perform a two-party protocol, in which they securely cluster the staff members by means of the frequency of their patient facing times. We describe the secure solution in detail, and evaluate the performance of our proof-of-concept. This work thus demonstrates the feasibility of secure multi-party clustering in this setting.

## Introduction

Hospitals are highly complex organizations typically involving a toxic combination of unpredictable patient flows and limited staffing and equipment resources. Achieving the Quadruple Aim (which aims to simultaneously improve Patient Experience, Population Health, Cost of Care and Provider Well-Being [28]) under such challenging conditions, often drives senior healthcare management to find every opportunity to optimize resources within the hospital.

A common approach taken by hospitals to optimize workflows is to hire consultants who interview and shadow key stakeholders and patients in order to develop an accurate picture of how the targeted department/hospital is functioning. A well-known drawback of such an approach is that individuals tend to change their behavior due to their awareness of being observed (a phenomenon known as the Hawthorne effect [36]). In addition, such manual observations only allow for point measurements, as it is impossible for any group of visiting consultants to accurately capture the operational characteristics of all key individuals in a department at any given time. Interviews are also unable to accurately capture data, as people often report their perception of events rather than facts.

Some hospitals approach this problem with a data-driven strategy. This involves going through the time-stamps entered in various hospital IT systems, e.g. in Electronic Health Record (EHR) systems, Staffing Information Systems, Laboratory Information systems, etc. While this is a better strategy than simply depending on manual observations, the data entered into hospital IT systems is highly susceptible to data quality issues [9, 24, 34]. Optimizing hospital workflows based on such noisy data can lead to erroneous outcomes [37].

One option is to use a Real-Time Locating System (RTLS) to help address the problem of inaccurate time-stamps. An RTLS consists of tags that can be placed on patients, staff and assets. The tags allow the locations of all tagged entities to be tracked at high spatial and temporal (e.g. every few seconds) resolution throughout the defined area of interest (e.g. within a hospital department). The real-time streaming data can also be used to automatically and accurately label many events. For example, a tagged patient would allow the system to accurately label when a patient has moved into a particular exam room. Similarly, a tagged nurse could be used to determine how many times the nurse has moved back and forth between two rooms of interest. Patient and staff location information can then be combined and plugged into certain common data mining algorithms (e.g. *k*-means clustering, sequential pattern analysis, or market basket analysis) to analyze the utilization patterns of various hospital rooms and to highlight any abnormalities that might exist. Such information can subsequently be used to identify bottlenecks and thus optimize workflows.

Under current hospital practices, hospitals routinely monitor staffing logs which describe which members of staff are on duty at any point in time; such information is critical for running a hospital. However, fine-grained location data of staff members is not currently considered routine in hospitals. Moreover, location data is considered as personal data in Europe under the newly established GDPR. This means that it is essential for a hospital to be completely transparent about what data is collected about individuals and gather permission from them prior to collecting and using the data; on the other hand, in order to perform effective and accurate workflow analysis based on location data, it is essential to have a high degree of participation from staff members. With hospital boards under constant pressure to improve productivity, sharing real-time location data of staff members with higher management could be considered to be a step too far. Such fear could greatly limit the number of participants who agree to sharing their location information. Moreover, privacy regulations such as GDPR, when it comes to dealing with patient records, mean that hospitals are not allowed to send any data beyond their physical boundaries.

A traditional approach in this case would be for the hospital to hire a trusted third party that collects all RTLS data, and outputs the clustering results. This party would be obliged, by contract and law only, not to disclose the RTLS data. However, the especially sensitive type of data involved would require expensive security measures. Furthermore, having all data at one single place increases the risk of information leakage. This makes it highly challenging to perform any kind of workflow optimization by analyzing these separate patient and staff RTLS data streams jointly.

In order to address this problem, this exploratory paper demonstrates how Secure Multi-Party Computation (shortened as MPC) can be used to allow data mining algorithms, such as *k*-means clustering, to be performed on two separate RTLS data streams: one generated by tagged patients, and the other by tagged hospital staff, while maintaining the privacy of all individuals. The location information of patients will only be made accessible to the hospital, while the location information of staff members will only be accessible to the staff members themselves, or to the labour union that represents them; labour unions, having the goal to represent the interests of all staff members of the hospital, are effectively the only body that can collectively act on behalf of all the nurses in a hospital.^1^ By splitting sensitive location information into two parts (patients and staff), each part being handled by a suitable independent party (hospital and labour union), we avoid any party gaining location information that they are not supposed to learn. Such a scheme allows the hospital to derive insights using both patient and staff RTLS data streams, without having access to individual location data streams of its staff members. The labour union makes secondary use of location data of its members (i.e., the hospital staff) impossible.

More concretely, we show the feasibility of this approach with a demonstrator that clusters nurses based on their patient facing time. This is motivated by the fact that hospital departments generally have some expectations in terms of how they should operate: for instance, in a hospital ward, patients typically arrive from different parts of the hospital with medical conditions of various type and of various degree of seriousness. As a consequence, nurses may be given different tasks and be requested to assist patients of a given ‘type’, where a type can indicate the medical condition of a patient or its seriousness. Clustering nurses, i.e. assigning them to separate sets based on the frequency and duration of interactions with patients of different types, can assist hospitals in determining whether nurses are indeed behaving as expected. Unexpected behavior may be a sign of sub-optimal workflow (e.g., signaling how other tasks prevent nurses from focusing on the assigned patients), and may thus lead to further investigation on the part of the hospital. For the proof-of-concept described in this article, we focused on *k*-means clustering, due to its popularity and its relative conceptual simplicity; *k*-means clustering is commonly used, for instance, when performing workflow analysis [23, 29, 35].

We stress the fact that the usage of RTLS in this setting is still in its infancy, and precise requirements are thus yet to be determined; in particular, it is still unclear at this point which data analysis algorithm can give the best insight in hospital workflow. We believe that the solution we present could also potentially help in clarifying needs and goals for an RTLS-based hospital workflow analysis, with *k*-means clustering of nurses based on patient facing times constituting a first use-case.

In the remainder of this section, we introduce the concept of secure multi-party computation, and give an overview of related work. In “Details of the computation” the details of all computational steps are explained, and in “Secure solution” it is shown how these could be performed securely. The performance results are shown in “Implementation and results”, and we end with the conclusions.

### Secure multi-party computation

The idea of MPC is that different mutually-distrusting parties compute the output of a certain function or computation, depending on private inputs of each party, without actually revealing information on their inputs. MPC has been introduced by Yao in the 1980s [39], and has led to a new flourishing research area yielding secure solutions for a large number of applications. Although efficiency was often a bottleneck, various implementation frameworks for MPC have appeared, especially during recent years, incorporating the latest technical accelerations, bringing applications towards practice [25].

To illustrate how the seemingly impossible requirements of MPC can be met, we briefly discuss a paradigm for constructing MPC protocols, which is widely used by the most recent generation of MPC frameworks. This paradigm is referred to as *share-compute-reveal*, and works in three phases: first, the input data is ‘secret-shared’ between the different parties, then a secure computation of the function is performed, and finally the output is revealed to the authorized party. All sensitive (intermediate) values are secret-shared, which means that each party obtains a non-revealing part of the data, called *share*, and the actual secret can only be obtained after combining all shares.^2^ Therefore, the data is secure as long as not all parties collude, and the parties can securely compute the desired function with sensitive information. Once the output has been securely computed, the parties can jointly reveal it; this means that the output of the computation is the only information learned by the parties.

Various applications of MPC in the medical domain have been presented, e.g., privacy-preserving data mining for joint data analysis between hospitals [26], branching programs for privacy-preserving classification of medical ElectroCardioGram signals [7], and also secure disclosure of patient data for disease surveillance [20], R-based healthcare statistics [15], and privacy-preserving genome-wide association studies [11].

### Related work

The potential benefits derived from using real-time locating systems in hospitals and other healthcare facilities have been presented in several papers [6, 8, 19, 30]. The security and privacy implications of pervasive data analysis techniques for healthcare, moreover, are widely discussed in the scientific community; see e.g. [1, 2] for some surveys on the topic.

To the best of our knowledge, this is the first paper that studies the usage of MPC for secure hospital optimization. However, other privacy-preserving techniques for healthcare data analysis have been presented in [32], and several MPC techniques for secure data analysis, and clustering in particular, have been presented in the past few years [3, 4, 10, 14, 21, 22, 27]. These MPC-based works differ from our approach in that they are set in the so-called ‘honest-but-curious’ model, where security is only guaranteed as long as parties follow the instructions of the protocol, while our solution is also secure in the ‘malicious’ model where one (or several) parties deviate from the instructions of the protocol.

Another important difference is that previous works on secure clustering assume that data is partitioned between parties, either horizontally (meaning that different data points will be owned by different parties) or vertically (meaning that each party only holds specific attributes of any data point). Our assumptions and requirements are different, as the data to be clustered is sensitive information that should remain hidden from *both* parties; a securely-distributed version of it — or, formally, a secret-shared version of it — is thus constructed in a first step of our solution (cf. “Secure solution” for details). Although showing the feasibility of secure clustering for hospital optimization is the main contribution of this manuscript, we thus believe the secure-clustering protocol itself to be of independent interest.

Unlike some of the related work mentioned above, we use secret sharing instead of (additive) homomorphic encryption. The main disadvantage of homomorphic encryption is that it leads to big overheads, because cipher texts need to be large for security reasons, which induces considerable computational efforts, and large amounts of communication. On the other hand, secret shares can be much shorter, and secure frameworks based on them (see “The MPC framework of our choice: SPDZ”) have been recently developed, which are quite efficient.

## Details of the computation

In this section we give a precise description of the algorithm that we wish to compute. We stress the fact that what is described here is the ‘plaintext’, or ‘unsafe’ computation, where privacy-sensitive data of patients and staff members is used. We show in “Secure solution” how to securely compute the functionality described in this section.

As informally described in the introduction, the input of the clustering algorithm is given by the RTLS data, which gives a snapshot of the hospital every few seconds, identifying where each patient and each staff member is at a given moment. The algorithm uses this input to cluster nurses according to the frequency and length of interactions with patients (the so-called *nurse-patient facing time*). Focusing on this concrete use-case, we will henceforth speak of ‘nurses’ instead of more generic ‘staff members’.

In order to realize this functionality, we developed a two-step algorithm: first, we construct a table that combines the RTLS data from the hospital and the labour union, and secondly, *k*-means clustering is applied to this table.

We describe the two parts of the computation in more detail in the following sections. The parameters used in the computation are listed in Table 1.

**Table 1:** Parameters

Parameter	Description
Nn	number of nurses
Np	number of patients
Nptype	number of patient types
nID	nurse ID
tagID	tag ID
zID	zone ID
time	time record
tagRole	person role tag
nP	set of nurse periods
pP	set of patient periods
st	starting time of a period
et	end time of a period
Ntimebins	number of time bins
TB	array with time bin boundaries
ov	overlap between interaction periods
ovbin	time bin indicator of overlapping periods

### Constructing the table

Since the hospital and the labour union each own a part of the RTLS data, which is needed to determine and compare the behaviour of the nurses, the first step of the computation is to combine these data. The outcome of this step is a table that associates each nurse to an array, indicating frequency and length of her/his interactions with patients, which can be used as input for a clustering algorithm.

As mentioned in the introduction, both the hospital and the labour union receive RTLS data, which consists of a series of rows formatted as defined in Table 2.

**Table 2:** Structure of raw RTLS data

Tag	Role	Time stamp	Zone
tagID_1_	tagRole_1_	time_1_	zID_1_
tagID_2_	tagRole_2_	time_2_	zID_2_
$\dots $	$\dots $	$\dots $	$\dots $

The tag tagID is the unique identifier assigned to each tag, while the role tagRole defines whether the tag belongs to a nurse or a patient; as stated in the introduction, what is crucial for the privacy of our solution is that the hospital will receive only rows with tag roles for *patients*, and the labour union will receive only rows with tag role *‘nurse’*.

The tag role also serves another goal, namely, it differentiates between various patient types. Indeed, patients are divided into Nptype ‘types’, according to the nature and severity of their medical condition; types could thus denote, for instance, terminally ill patients, or patients suffering from a heart attack. Each row of the table means that the individual with tag tagID was in a zone with identifier zID at time time, where tagRole gives additional information on the individual (role and patient type, if applicable).

As a preliminary step, both the hospital and the labour union locally pre-process their RTLS data. The goal of this pre-processing is to obtain for each nurse (resp. patient) what we call his/her period data, where *periods* are continuous stretches of time where the nurse (resp. patient) remained in one zone. Formally, period data is formatted as in Table 3, where each row means that a nurse (resp. patient) with tag tagID, and with tag role tagRole, remained in zone zID from time st to time et. In general, there will be several rows with the same tagID, since patients and nurses move around the hospital, and the table of the hospital (resp. labour union) will only contain rows corresponding to patients (resp. nurses), as they only have access to RTLS data of this type.

**Table 3:** Structure of individual pre-processed RTLS data

Tag	Start time	End time	Zone	Role
tagID_1_	st_1_	et_1_	zID_1_	tagRole_1_
tagID_2_	st_2_	et_2_	zID_2_	tagRole_2_
$\dots $	$\dots $	$\dots $	$\dots $	$\dots $

Following this pre-processing, the hospital and the labour union collaborate with each other in order to obtain a shared table, which assigns to each nurse an array indicating how many interactions of a given length the nurse had with patients of given type (cf. Table 4).

**Table 4:** Nurse-patient facing times

	Patient Type A				Patient Type B			
nID	0-10	10-30	30-60	> 60	0-10	10-30	30-60	> 60
nID_1_	⋆	⋆	⋆	⋆	⋆	⋆	⋆	⋆
nID_2_	⋆	⋆	⋆	⋆	⋆	⋆	⋆	⋆
$\dots $	$\dots $	$\dots $	$\dots $	$\dots $	$\dots $	$\dots $	$\dots $	$\dots $

Notice that for simplicity, Table 4 only shows two patient types (‘A’ and ‘B’); entries denoted by ⋆ are aggregates, indicating how many times the nurse nID_*i*_ was in the same zone as a patient of the specified type (‘A’ or ‘B’) for a period of time within the specified ‘time-bin’ (less than 10 seconds, between 10 and 30, and so on).

Algorithm 1 specifies how to compute Table 4 from the two tables of patient/nurse data owned by the hospital and the labour union. In Algorithm 1, pP indicates the number of patient periods, i.e., the number of rows of the table owned by the hospital (cf. Table 3). For each $i=1,\dots ,\textsf {pP}$, we denote the *i*-th row of the table owned by the hospital by $(\dots , \textsf {st}_{i}, \textsf {et}_{i}, \textsf {zID}_{i}, \dots )$, and similarly for the nurse data owned by the labour union. 
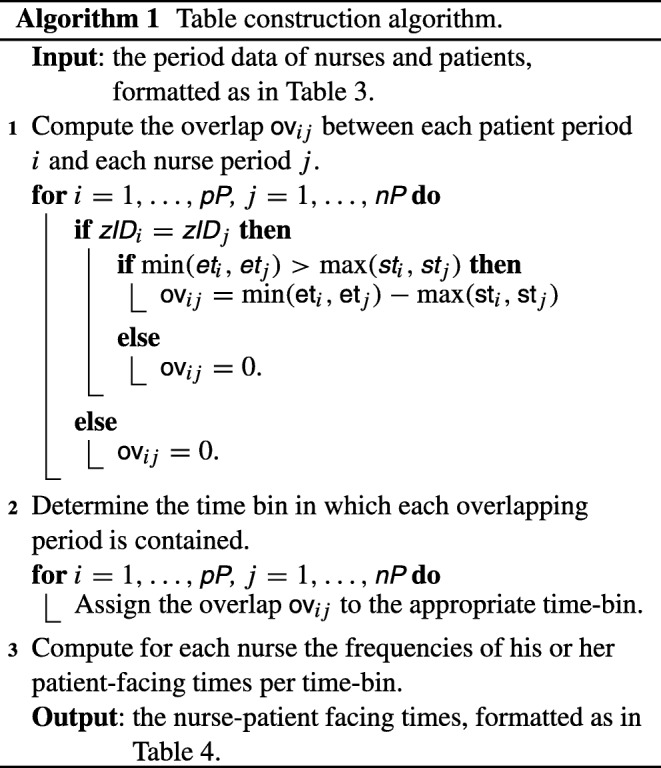


### K-means clustering

The computation described above associates each nurse to an array of non-negative integers, where each entry specifies how many interactions of a given length the nurse had with patients of given types (cf. Table 4).

*Clustering,* a branch of unsupervised machine learning, offers a way to extract valuable information from this data: informally speaking, it allows us to find a partition of the set of nurses into disjoint sets, or *clusters*, in such a way that ‘similar’ nurses (i.e., with a ‘similar’ associated array) belong to the same cluster, while ‘dissimilar’ nurses belong to different clusters.

We focus on *k-means* clustering, widely used due to its relative simplicity and applicability to large data sets [33]. The *k*-means algorithm works as follows: denote by $\mathbf {y}^{(i)}\in \mathbb {R}^{{m}}$ (where *m* = Ntimebins ⋅Nptype) the vector, or *data point,* associated with the *i*-th nurse for every $i=1,\dots ,\textsf {Nn}$, and let $\mathcal {S}$ denote the list $(\mathbf {y}^{(1)},\dots ,\mathbf {y}^{(\textsf {Nn})})$ (i.e., the list consisting of the rows of Table 4). While various notions of similarity between data points can be defined, *k*-means clustering typically assumes that a distance *d* is defined over the vector space the data points belong to; we assume for simplicity that *d* is the Euclidean distance, which is the most common case in *k*-means clustering.

Formally, the goal of *k*-means clustering is to find a partition $(\mathcal {S}_{1}, \dots , \mathcal {S}_{k})$ of the list $\mathcal {S}$ of data points, i.e., $\mathcal {S} = \mathcal {S}_{1} \sqcup {\dots } \sqcup \mathcal {S}_{k}$, so as to minimize the quantity ${\sum }_{j=1}^{k} {\sum }_{\mathbf {y} \in \mathcal {S}_{j}}d(\mathbf {y},\boldsymbol {\mu }_{j})^{2}$, where ***μ***_*j*_ denotes the arithmetic mean of the points belonging to the *j*-th cluster.

Exact *k*-means clustering is, in fact, an NP-hard problem [33]; for this reason, an approximate iterative algorithm sometimes called *Lloyd’s algorithm,* presented below in Algorithm 2, is typically used instead. This algorithm is so ubiquitous that it is often referred to as *the* *k*-means clustering algorithm, a convention that we will also adopt. 
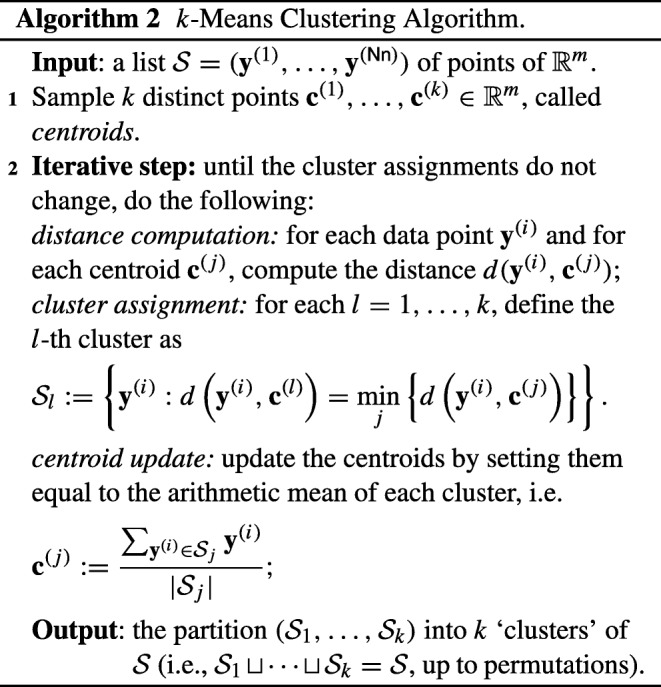


The output of Algorithm 2 does not encompass the centroid values: this is due to our MPC-motivated approach, since the centroids may reveal sensitive information. We also remark that the description of Algorithm 2 only provides a skeleton of the actual *k*-means algorithm, as it does not specify how to sample the initial centroids, and does not handle some degenerate cases which make the algorithm ill-defined (notably, it implicitly assumes that clusters are never empty). Several approaches are possible to fill these gaps and obtain a fully-fledged specification of *k*-means; in the following section, we will detail the solution of our choice, highlighting the reasons that led us to select them.

## Secure solution

In order to develop a secure solution, we make use of MPC schemes based on so-called *secret sharing* techniques. The owner of each entry *x* of Table 3 uploads this entry as a secret, shared between the hospital and the labour union. We denote the resulting secret-shared value by $\left \langle x \right \rangle $; such a secret-shared value consists of two shares, *x*_1_ and *x*_2_, held by the hospital and the labour union respectively. The fundamental property of this secret-sharing process is that a single share *x*_*i*_ gives no information whatsoever on the original value *x*, but the two parties can cooperate to perform computations on secret-shared data and, if required, jointly reconstruct the value of a secret-shared element.

The secret-sharing-based framework of our choice, SPDZ (cf. “The MPC framework of our choice: SPDZ”), ensures that our solution is secure under the assumption that the involved parties are restricted to polynomial-time computation, and safeguards the privacy of each party’s input and the correctness of the result even if one of the parties actively cheats and does not follow the instructions of the protocol.^3^

In our setting (cf. “Details of the computation”), the secure computation on the secret-shared data consists of two parts, each being explained in more detail further on:

A. A secure computation of the table consisting of facing times frequencies per nurse (see Table 4).
B. A secure clustering of the nurses, based on this table.

Prior to the secure computation of the table, both parties need to locally transform their RTLS data into a series of time intervals per zone (see also “Constructing the table”), as illustrated in Table 3. Since this does not require combining data of patients and nurses, there is no security issue: parties can perform this processing locally, and we therefore do not further discuss this preliminary step.

### Secure table construction

The input of the first step of the computation is given by a secure variant of Table 3, where all entries have been secret-shared between the two parties. In order to obtain a table of nurse-patient facing times, we need to translate Algorithm 1 to the encrypted domain — namely, we need to specify how all steps of Algorithm 1 can be performed on secret-shared data.

As a first step, we discuss the translation to the encrypted domain of basic operations:

*Sum and multiplication:* these can be directly computed on secret-shared inputs by secret-sharing based MPC protocols [12]. The same also holds with addition and sum between a secret-shared input and a public constant.
*Secure comparison:* securely checking whether *a* < *b* for secret-shared values $\left \langle a \right \rangle $ and $\left \langle b \right \rangle $ can be performed by any secure comparison protocol [12], given the above basic operations. We do not describe here how this is exactly performed by an MPC protocol, and denote the output of a secure comparison as follows:
  $$ \langle (a \overset{?}{<} b) \rangle \text{, where } (a \overset{?}{<} b) = \left\{\begin{array}{ll} 1 & \text{, if } a<b, \\ 0 & \text{, otherwise}. \end{array}\right. $$
  Similarly, one can securely compute a secret-shared bit $\langle (a\overset {?}{\geq } b) \rangle $ that expresses whether *a* ≥ *b*, or not.
*Minimum and maximum computation:* given a secret-sharing of $\epsilon = (a \overset {?}{<} b)$, the minimum (resp. maximum) between two secret-shared values *a* and *b* can be readily computed by means of the above operations:
  $$ \left\langle \min(a,b) \right\rangle = \left\langle a \right\rangle \cdot \langle \epsilon \rangle + \left\langle b \right\rangle \cdot \left( 1 - \langle \epsilon \rangle \right), $$
  and similarly for the secure maximum function.

With these building blocks in place, Algorithm 1 can be translated to the secure domain; the overall description can be found in Algorithm 3. 
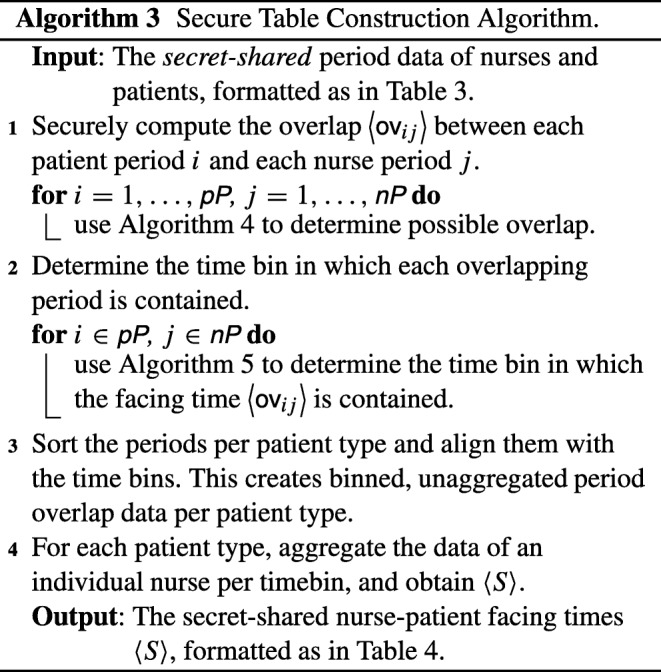
 
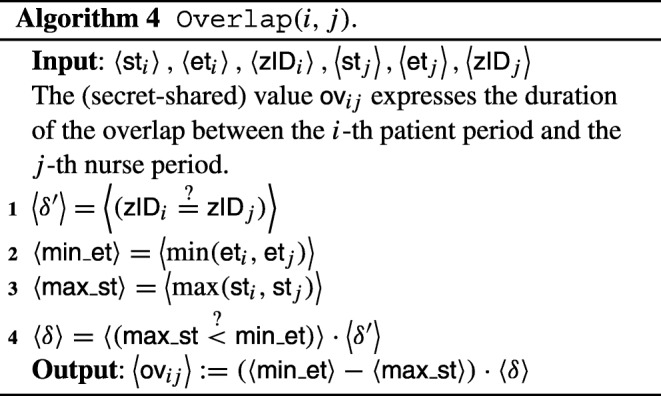
 
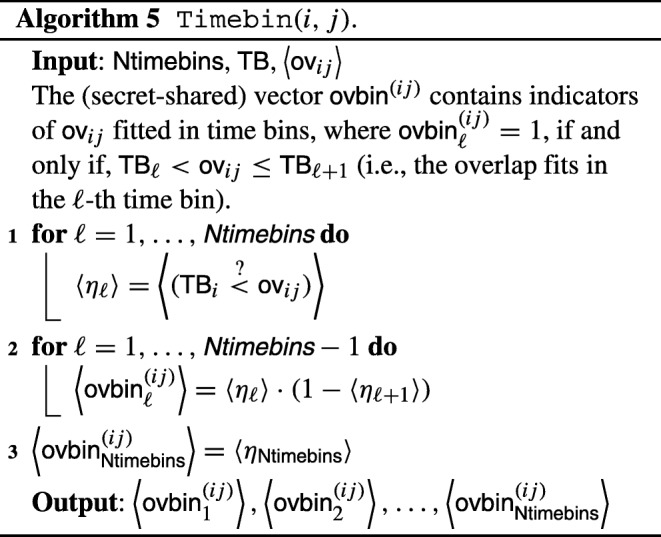


### Secure *k* −means clustering

After the above step has been performed, we thus obtain a (secret-shared) table that associates to each nurse a secret-shared data point (vector) $\left \langle \mathbf {y}^{(i)} \right \rangle $ expressing how many interaction periods of a given length, and with a patient of given type, the *i*-th nurse had.

To perform secure *k*-means clustering over secret-shared data, we construct a *membership matrix* $\textbf {M} \in \mathbb {N}^{\textsf {Nn} \times {k}}$, where **M**_*ij*_ = 1, if the *i*-th data point belongs to the *j*-th cluster, and **M**_*ij*_ = 0, otherwise. The idea is then to keep **M** secret-shared, and only to reveal it at the last step of the clustering algorithm.

With this concept in mind, one can then transpose the ‘skeleton’ *k*-means Algorithm 2 to an MPC setting: the key points of the iterative steps are presented below.

*Distance computation:* since sums and multiplications can be directly computed, we can securely compute the (secret-shared) value
  $$ \left\langle d^{2}\left( \mathbf{y}^{(i)},\mathbf{c}^{(j)} \right) \right\rangle = {\sum}_{\ell=1,\dots,{m}} \left( \left\langle \mathbf{y}^{(i)}_{\ell} \right\rangle - \left\langle \mathbf{c}^{(j)}_{\ell} \right\rangle \right)^{2} $$
  for each nurse **y**^(*i*)^ and each cluster (with centroid) **c**^(*j*)^.
*Cluster assignment:* by making use of a secure-comparison subroutine as described in the previous sub-section, we can compute for any $\mathbf {y}^{(i)},\mathbf {c}^{(j)},\mathbf {c}^{(j^{\prime })}$ the following secret-shared value:
  $$ \left\langle \xi(i,j,j^{\prime}) \right\rangle:=\left\langle \left( d^{2}\left( \mathbf{y}^{(i)}, \mathbf{c}^{(j^{\prime})}\right) \overset{?}{\geq} d^{2}\left( \mathbf{y}^{(i)},\mathbf{c}^{(j)} \right) \right) \right\rangle $$
  We can then set $\left \langle \textbf {M}_{ij} \right \rangle = {\prod }_{j^{\prime }=1}^{k} \left \langle \xi (i,j,j^{\prime }) \right \rangle $.
*Centroid update:* Assuming the selected MPC protocol has a built-in secure integer-division subroutine (for fixed- or floating-point numbers), we can securely compute the value
  $$ \left\langle \mathbf{c}^{(j)} \right\rangle = \frac{{\sum}_{i=1}^{\textsf{Nn}} \left\langle \textbf{M}_{ij} \right\rangle \cdot \left\langle \mathbf{y}^{(i)} \right\rangle}{\left\langle {\sum}_{i=1}^{\textsf{Nn}} \textbf{M}_{ij} \right\rangle} $$
  for all $j=1,\dots ,{k}$.
  At the end of this section, we show how to avoid this expensive subroutine, and only use basic operations and comparisons instead.

In order to obtain a fully-fledged secure *k*-means algorithm, however, we had to address the following remaining points:

1. A method to sample the *k* initial centroids needs to be specified;
2. The algorithm does not prevent assignment of a data point to several clusters. It would be preferable to assign each point to one cluster only;
3. If a cluster becomes empty, then the algorithm is ill-defined, as it attempts to perform a division by $|\mathcal {S}_{j}|= {\sum }_{i} \textbf {M}_{ij} =0$. A method to prevent this should be specified;
4. A routine that checks whether the algorithm has converged (i.e., whether the cluster assignment did not change at the last iteration) should be specified.

We now describe our solution to the above issues. Furthermore, we show how we can avoid expensive fixed- or floating-point computation and restrict ourselves to more efficient integer arithmetic.

#### Sampling initial centroids

Various methods are used in standard *k*-means clustering to sample the initial centroids, often selecting them among the data points via a randomized choice method. While more involved techniques such as *k*-means++ [5] can guarantee faster convergence and/or better cluster quality, we opt for a simpler method, which can very efficiently be implemented in a secure way, and which is sufficient for our goal of showing the feasibility of an MPC solution. We thus select the initial centroids by sampling *k* elements among the data points; this random sampling can be executed, for instance, by the hospital, who should be in charge of the decision of the relevant clustering parameters, given that it is the entity interested in the workflow analysis.

#### Avoiding multiple assignment

As we noticed above, if for a given data point **y**^(*i*)^ there are two centroids $\mathbf {c}^{(j_{1})},\mathbf {c}^{(j_{2})}$ such that $d(\mathbf {y}^{(i)},\mathbf {c}^{(j_{1})}) = d(\mathbf {y}^{(i)},\mathbf {c}^{(j_{2})}) = \min \limits _{j} (d(\mathbf {y}^{(i)},\mathbf {c}^{(j)}))$, then the algorithm sets $\textbf {M}_{ij_{1}}=\textbf {M}_{ij_{2}}=1$. It would instead be desirable to assign **y**^(*i*)^ to a unique cluster. In order to do this, we simply assign **y**^(*i*)^ to the cluster with the lowest index; this can be done securely by setting **M**_*ij*_ = 0, if $\textbf {M}_{ij} \leq \textbf {M}_{ij^{\prime }}$ for some $j^{\prime }<j$, which can be done via a secure-comparison subroutine.

#### Handling empty clusters

As highlighted above, Algorithm 2 is not guaranteed to be well-defined. Namely, if a cluster becomes empty, the algorithm will attempt to divide by 0 upon computing the new centroid corresponding to that cluster. Once again, several methods are used in (non-secure) *k*-means clustering to address this problem. Most of these methods take action in case an empty cluster is detected, for instance by assigning a given data point to an empty cluster. This is arguably a sub-optimal approach in secure *k*-means, since it either requires revealing intermediate cluster assignments (which could undermine the security of our solution), or it can lead to increased complexity by checking in a secure way whether there is an empty cluster. We adopt an alternative approach, described in [31]: simply add each centroid to its corresponding cluster. As shown in [31], the convergence time of the algorithm is only slightly increased with this method.

#### Adding a convergence check

As a general rule, the *k*-means algorithm is supposed to stop only after it has converged, i.e., once the cluster assignments (and centroid values) no longer change. Such a check can be performed in an (almost) oblivious way by means of secure equality; we stress the fact that this is a relatively expensive check, and we thus prefer not to execute it after every iteration of the algorithm. A better alternative is to only run it after the last iteration, or, alternatively, after any fixed number of iterations. In our simulations, we made use of the first alternative.

Altogether, the above sub-routines yield a complete specification of a circuit modeling secure *k*-means clustering.

#### Improving Efficiency with Integer-Only Computation

An important remark to improve the efficiency of our solution is that the data points $\mathbf {y}^{(1)},\dots ,\mathbf {y}^{(\textsf {Nn})}$ of nurse-patient interaction periods are vectors with *integer-only* entries. We can exploit this fact designing a centroid-update routine that only makes use of secure integer arithmetic (instead of fixed- or floating-point), significantly improving the efficiency of secure *k*-means clustering. Notice that integer arithmetic can be readily simulated by choosing a large enough integer *M* and then embedding $\mathbb {Z}\cap [-M,M]$ into a prime field $\mathbb {F}_{p}$ for any *p* > 2*M*; in contrast, simulating fixed- and floating-point arithmetic in a finite field is a more involved and computationally-expensive process.

First of all, since $\mathbf {y}^{(i)}\in \mathbb {N}^{{m}}$ for all *i*, then each centroid **c** will be of the form $(\mathbf {x}_{1}/{w},\dots ,\mathbf {x}_{{m}}/{w})$, where $\mathbf {x}_{i},{w}\in \mathbb {N}$. Thus for any two centroids $\mathbf {c}=(\mathbf {x}_{1}/{w},\dots ,\mathbf {x}_{{m}}/{w})$, $\tilde {\mathbf {c}}=(\tilde {\mathbf {x}}_{1}/\tilde {{w}},\dots ,\tilde {\mathbf {x}}_{{m}}/\tilde {{w}})$ and any point **y**, we have that $d^{2}\left (\mathbf {y},\mathbf {c} \right ) \leq d^{2}\left (\mathbf {y}, \tilde {\mathbf {c}} \right )$, if and only if, the following holds:

$$ \begin{array}{@{}rcl@{}} &&\sum\limits_{i} \left( \mathbf{y}_{i}- \frac{\mathbf{x}_{i}}{{w}} \right)^{2} \leq \sum\limits_{i} \left( \mathbf{y}_{i}- \frac{\tilde{\mathbf{x}}_{i}}{\tilde{{w}}} \right)^{2} \\ &\iff & \sum\limits_{i} \left( \frac{{\mathbf{x}_{i}^{2}}}{{w}^{2}} - 2\frac{\mathbf{x}_{i}}{{w}} \mathbf{y}_{i} \right) \leq \sum\limits_{i} \left( \frac{\tilde{\mathbf{x}}_{i}^{2}}{\tilde{{w}}^{2}} - 2\frac{\tilde{\mathbf{x}}_{i}}{\tilde{{w}}} \mathbf{y}_{i} \right) \\ &\iff & \tilde{{w}}^{2}\sum\limits_{i} \left( {\mathbf{x}_{i}^{2}} - 2{w}\mathbf{x}_{i} \mathbf{y}_{i} \right) \leq {w}^{2}\sum\limits_{i} \left( \tilde{\mathbf{x}}_{i}^{2} - 2\tilde{{w}}\tilde{\mathbf{x}}_{i} \mathbf{y}_{i} \right). \end{array} $$

This means that the distance-comparison of the *k*-means algorithm can be performed with simple *integer* arithmetic, instead of fixed- or floating-point arithmetic.

The above steps thus form a fully-fledged and efficient secure *k*-means clustering algorithm, which we believe to be of independent interest as well.

## Implementation and results

We describe in this section our implementation of the secure solution of “Secure solution”, and present some evaluation of its performance.

### The MPC framework of our choice: SPDZ

We chose to use SPDZ [17, 18], a recent secret-sharing-based MPC platform of celebrated efficiency. A software suite for UNIX systems based on the SPDZ platform is publicly available [13, 16];^4^ we used this suite to implement our secure solution for workflow analysis.

SPDZ has built-in functionalities for secure comparison, and can thus be used to implement the building blocks described in “Secure table construction”. SPDZ needs to produce some raw material in a pre-computation phase in order to securely evaluate these functionalities; however, this pre-computation is independent of the actual function to be computed and of the secret inputs, and can thus be executed on idle time between the two parties. For this reason, we neglect pre-processing when measuring the performance of our solution.

### Set-up

In order to test the efficiency of the algorithms we developed, we ran several simulations on two physically-separated machines, representing the hospital and the labour union, respectively. Both machines were equipped with of a 3.5 GHz Intel i7-7567U CPU and 32 GB of RAM, and were connected to each other via a 1 Gbit/s wired network. Furthermore, the SPDZ protocol has been instantiated with 40-bit statistical security, 128-bit computational security and a 64-bit prime field.

### Performance results

Several simulations were run in order to measure the efficiency and scalability of both phases of our secure solution in the above set-up. We sampled artificial data for these simulations, made to resemble a realistic size of a hospital department and realistic behavior of nurses [38]: we considered a fixed number of 15 zones and a total study time of one hour, in which tracking information was produced every 4 seconds. We assumed that nurses remain in the same zone for up to 120 seconds, while patients can remain in the same zone for the entire hour. Accordingly, we considered 4 time bins, namely 0-to-10 seconds, 10-to-30 seconds, 30-to-60 seconds, and more than 60 seconds.

We measured the elapsed computation time and the communication cost while varying either the number of patient types (3, 5, 10), considering a fixed number of 7 patients per patient type, or the number of nurses (5, 12, 30, 60, 120). We also investigated the effect of increasing the total number of clusters, considering 2, 5 and 10 clusters, while fixing at 5 the number of iterations of the *k*-means clustering protocol.

**Fig. 1:**
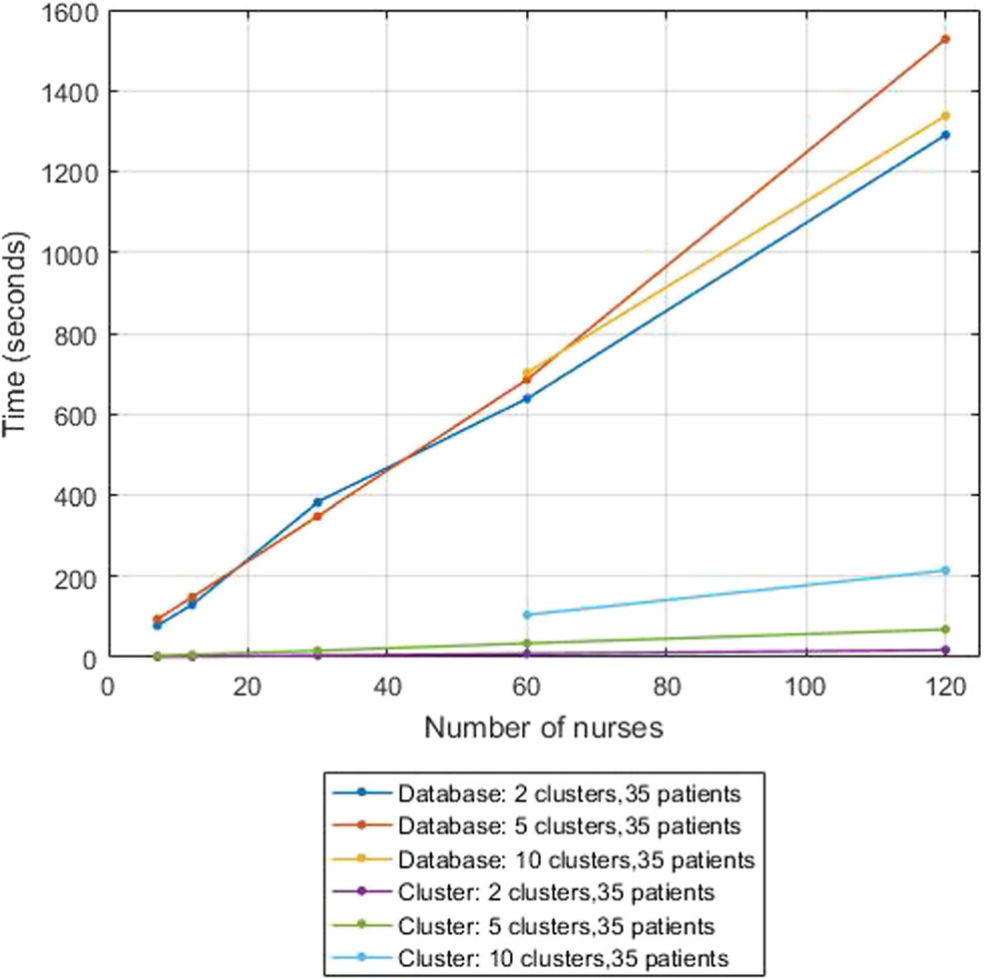
Computation time (5 iterations), varying the number of nurses

**Fig. 2:**
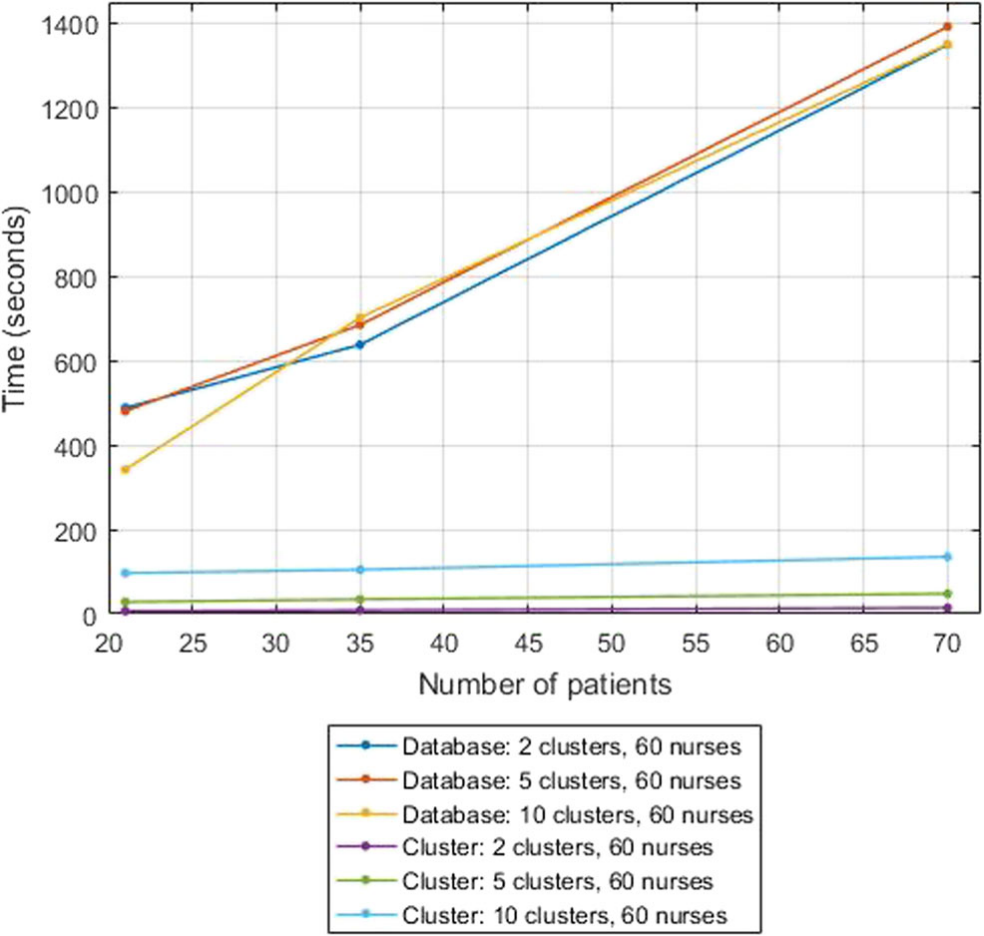
Computation time (5 iterations), varying the number of patients

We measure the computation time of the two phases of the secure protocol separately. It is clear from Figs. 1 and 2 that the first phase, the database construction, is more computationally-intensive than the second phase, the *k*-means clustering (with 5 iterations). Notice that the computational cost of the second phase increases linearly in the number of iterations.

In Fig. 1 we varied the number of nurses, while fixing at 5 the number of patient types. We observe that the computation time of the first phase grows linearly in the number of nurses; this matches our expectations, since theoretically the complexity of this phase scales linearly with the number of nurse time periods, which in turn grows linearly with the number of nurses in our simulations. Also notice that the computation time of this phase is independent of the number of clusters, as this number only plays a role in the second phase of the protocol. Further, for each experiment, the total number of patient time periods varies, as for each experiment new artificial data is generated; this explains the slight variation in the timing results of the first phase. Furthermore, the timing results indicate that the computation time of the second phase scales linearly in the number of clusters and in the number of nurses.

In Fig. 2 we varied the number of patient types, keeping the number of nurses fixed at 60. We note that the computation time of the first phase grows linearly with the total number of patients, again slightly fluctuating due to the fact that the total number of time periods (of patients and nurses) slightly varies per experiment. The computation time of the second phase scales linearly in the number of clusters and in the number of patient types.

Table 5 provides an overall view of the scalability of our solution, showing running time and total size of the data exchanged between the two parties, for a fixed choice of 5 clusters and for increasing numbers of nurses and patients.

**Table 5:** Runtime (seconds) and exchanged data (megabytes), 5 clusters

	**7 nurses**	**12 nurses**	**30 nurses**	**60 nurses**	**120 nurses**
**21 patients**	time: 108	time: 160	time: 310	time: 564	time: 1072
	comm.: 47	comm.: 95	comm.: 233	comm.: 499	comm.: 964
**35 patients**	time: 154	time: 212	time: 422	time: 816	time: 1677
	comm.: 90	comm.: 143	comm.: 335	comm.: 677	comm.: 1496
**70 patients**	time: 241	time: 384	time: 768	time: 1530	time: 2912
	comm.: 166	comm.: 297	comm.: 657	comm.: 1338	comm.: 2657

Notice that by inspecting the pseudo-code of our solution, it is readily seen that the observed linearity in the timing results is as expected. Finally, we note that the benchmarks described in this section are obtained with an implementation that still has plenty of room for efficiency improvement. Future development on this aspects could, for instance, benefit from further parallelization within both phases of the protocol, use of high-performance computing machines, or implementation in low-level, very fast programming languages such as C.

## Conclusion

We proposed a novel approach to analyze the joined location data of patients and staff in a hospital, by means of an innovative cryptographic technique called Secure Multi-Party Computation. In a joint protocol, the hospital and the labour union securely cluster the staff members by means of the frequency of their patient facing times.

In the first step, a table is securely constructed that contains for each nurse a secret frequency distribution of his, or her, patient facing times. In the second step, this table is used to cluster the nurses into similar groups. Although this secure *k*-means clustering algorithm is used for optimizing the workflow in a hospital, it could be used in many different domains where sensitive data needs to be clustered.

We described the secure protocol in detail, and evaluated its performance, thereby demonstrating the feasibility of our approach: it takes less than half an hour to securely cluster 120 nurses, who take care of 35 patients in 15 different zones, given location data of one hour and a tracking frequency of 4 seconds. While speed was not a factor of capital importance for our solution, given that data analysis does not need to be performed in real time, we believe that the good performance obtained by our protocol paves the way for more advanced data analysis techniques to optimize the workflow in a hospital.

Towards a fully operational deployment, however, some points need to be addressed. Notably, our solution was not tested on real data, given that even obtaining retrospective data would require individual consent from the involved staff members and patients; for operational deployment, however, this step will be necessary, in order to properly assess the impact of the data analysis. Moreover, while *k*-means clustering was a natural choice for a demonstrator due to its ubiquity and relative conceptual simplicity, several other machine-learning techniques could be securely implemented with our approach. This means that an appropriate evaluation and comparison of the various possibilities will have to be performed.

## Footnotes

1 We make the remark that our solution can also accommodate for the case of several labour unions, up to a natural extension of the steps described in “Constructing the table” and “Secure table construction”.

2 Thus ‘sharing’ is here by no means a synonym of ‘revealing’: secret-sharing can actually be seen as a strong form of distributed *encryption*.

3 In this case, however, it is not guaranteed that the honest party will obtain output: they might only detect that cheating occurred, and have at that point no other option than to abort the protocol.

4 Support for the SPDZ-2 implementation is being discontinued; development has shifted to the SCALE-MAMBA platform, which is also based on the SPDZ protocol.
